# A Physics-Informed Deep Learning Deformable Medical Image Registration Method Based on Neural ODEs

**DOI:** 10.1007/s11263-025-02476-6

**Published:** 2025-06-08

**Authors:** Amirhossein Amiri-Hezaveh, Shelly Tan, Qing Deng, David Umulis, Lauren Cunniff, Johannes Weickenmeier, Adrian Buganza Tepole

**Affiliations:** 1 School of Mechanical Engineering, Purdue University, West Lafayette, IN 47907 USA; 2 Department of Mechanical Engineering, Stevens Institute of Technology, Hoboken, NJ 07030 USA; 3 Department of Engineering Science, University of Oxford, Oxford, OX3 7DQ UK

**Keywords:** Deformable image registration, Physics-informed neural networks, Brain registration, Growth and remodeling, Brain development, Brain atrophy, Zebrafish biophysics, Neural ordinary differential equations, Medical image analysis

## Abstract

An unsupervised machine learning method is introduced to align medical images in the context of the large deformation elasticity coupled with growth and remodeling biophysics. The technique, which stems from the principle of minimum potential energy in solid mechanics, consists of two steps: Firstly, in the predictor step, the geometric registration is achieved by minimizing a loss function composed of a dissimilarity measure and a regularizing term. Secondly, the physics of the problem, including the equilibrium equations along with growth mechanics, are enforced in a corrector step by minimizing the potential energy corresponding to a Dirichlet problem, where the predictor solution defines the boundary condition and is maintained by distance functions. The features of the new solution procedure, as well as the nature of the registration problem, are highlighted by considering several examples. In particular, registration problems containing large non-uniform deformations caused by extension, shearing, and bending of multiply-connected regions are used as benchmarks. In addition, we analyzed a benchmark biological example (registration for brain data) to showcase that the new deep learning method competes with available methods in the literature. We then applied the method to various datasets. First, we analyze the regrowth of the zebrafish embryonic fin from confocal imaging data. Next, we evaluate the quality of the solution procedure for two examples related to the brain. For one, we apply the new method for 3D image registration of longitudinal magnetic resonance images of the brain to assess cerebral atrophy, where a first-order ODE describes the volume loss mechanism. For the other, we explore cortical expansion during early fetal brain development by coupling the elastic deformation with morphogenetic growth dynamics. The method and examples show the ability of our framework to attain high-quality registration and, concurrently, solve large deformation elasticity balance equations and growth and remodeling dynamics.

## Introduction

Image registration aims to obtain a smooth map that aligns the source and target images. There are two main categories of registrations: affine (or rigid) and deformable (non-rigid) registrations. In the earlier, the transformation consists of specific modes of deformations, including scaling, shearing, translation, and rotation. These transformations can be constructed using homogeneous coordinates and matrix transformation of size $4 \times 4$ and $3 \times 3$ for 3d and 2d images, respectively. Due to its finite parameters, this type of transformation is a good candidate when anatomical structures are mostly preserved during deformation. On the other hand, the latter is accompanied by more degrees of freedom, which can capture anatomical changes, such as embryo development and brain variations in different individuals, among other examples.

A subcategory of the non-rigid registration is physics-based models. Some of these models are based on the principles of continuum mechanics. The balance of internal and external forces dictates the transformation in this framework. The external forces are typically defined through similarity measures, e.g., cross-correlation of intensity of target and moving images (Pawar et al., 2018). The underlying balance laws are written either according to infinitesimal strain theory (Broit, 1981) or fluid flow algorithms (Christensen et al., 1994, 1996). The latter was specifically proposed to introduce large deformation theory in the context of the registration. In parallel, the utilization of nonlinear solid mechanics, which was manifested by using hyper-elastic constitutive equations and including nonlinear terms in the strain measure, can be seen, for instance, in Rabbitt et al. (1995), Pennec et al. (2005), Yanovsky et al. (2008), Pawar et al. (2022) (see more references in Sotiras et al. (2013)). As mentioned in Sotiras et al. (2013), from the biomedical application perspective, it is significant that the resulting transformation is invertible, and both the transformation and its inversion are differentiable (diffeomorphism). This group of transformations can be generated when the deformation is modeled as a (pseudo) flow, which is governed by the transport equation (Miller et al., 2002). This framework was originally proposed in the context of continuum flow algorithms (Christensen et al., 1996) and further developed in as large deformation diffeomorphic metric mapping (LDDMM) approaches (Joshi & Miller, 2000; Beg et al., 2005; Cao et al., 2005; Ceritoglu et al., 2009; Oishi et al., 2009; Hernandez et al., 2009; Ceritoglu et al., 2010), among others.

Apart from the above methods, the widespread use of deep learning methods in medical registration has been increasingly emerging. These methods can be mainly categorized into (Zou et al., 2022; Haskins et al., 2020) 1) deep interactive methods, 2) fully supervised methods, 3) unsupervised methods, and 4) weakly supervised. Among them, the development of unsupervised methods is significant due to the difficulty in providing a sufficient amount of ground truth data for the supervised methods (Zou et al., 2022). In the case of similarity-based unsupervised methods (Zou et al., 2022), the transformation is a neural network whose parameters are determined from the minimization of the loss function that consists of similarity measure and regularization term. De Vos et al. (2017) and Balakrishnan et al. (2019) are two examples of unsupervised learning approaches, among others (López et al., 2023). In contrast to classical methods, the registration in VoxelMorph is accomplished globally. That is, instead of aligning each pair of images separately, a global transformation is defined and parameterized by training a convolution neural network where the inputs are pairs of images. While this global training leads to a fast registration for unseen pairs of images, this method may not give good results for large deformations and is not guaranteed to provide diffeomorphic maps (Shen et al., 2019; Kuang & Schmah, 2019). However, there is a new trend to develop deep learning methods that learn diffeomorphic maps such as (Mok & Chung, 2020; Wang et al., 2024).

The resulting transformation of the classical frameworks lies in the solution of governing partial differential equations, which were mainly solved by means of numerical methods such as finite difference and finite element methods (FEM). However, the universal approximator property of feed-forward deep neural networks (Hornik et al., 1989) motivated researchers to employ neural networks as solvers for physical boundary value problems (so-called physics-informed neural networks (PINN)). Early attempts in this regard can be seen in Lagaris et al. (1997), Lagaris et al. (1998), Lagaris et al. (2000), McFall (2006), McFall and Mahan (2009). However, with the advent of libraries such as Tensorflow, Pytorch, and JAX, where the differentiation is computed through automatic differentiation in an efficient manner, there is a marked tendency to exploit deep neural networks as function approximators in various physical problems. These methods are mostly meshless, and there exist variants of it such as collocation (Sirignano & Spiliopoulos, 2018; Raissi et al., 2019), or integral forms (Kharazmi et al., 2019, 2021; Jagtap & Karniadakis, 2021; Rezaei et al., 2022). One long-standing problem with the meshless methods is the satisfaction of the Dirichlet boundary condition since the function expansion does not satisfy partial unity. In the classical version of PINN, these boundary conditions are enforced by adding an extra penalty term in the loss function, which leads to an inexact imposition of the boundary conditions. This approximation is negatively reflected in the accuracy of the solution (see (Sukumar & Srivastava, 2022)). Hence, Sukumar and Srivastava (2022), by using the approximate distance function— which can be generated from either theory of R functions (Rvachev, 1982; Biswas & Shapiro, 2004) or theory of mean value potential fields, defined a new format of solution and exactly fulfilled the displacement boundary conditions.

In this study, we propose a PINN-like approach in the context of large deformation solid mechanics for the registration of deformable (biomedical) images. The method consists of two parts: 1) predictor and 2) corrector. In the predictor part, the central focus is on obtaining a map that maximizes the similarity measure between moving and source images. To this end, we employ the following conventional multi-objective:

1.1 $$\begin{aligned} \mathop {\arg \,\min }\limits _\theta \left( {sim(T({\mathbf {\varphi }}({\textbf{X}};\theta )),I({\textbf{X}})) + \beta R({\mathbf {\varphi }}({\textbf{X}};\theta ))} \right) . \end{aligned}$$

in which *T* and *I* denote the target (moving) and initial (source) images, ${\mathbf {\varphi }}({\textbf{X}};\theta )$ is the corresponding map with parameters $\theta$ that aligns the target and initial images, *R* is a regularizing term, and $\beta$ is a relatively small positive hyper-parameter. It is noted that the terminology ’predictor’ is employed to emphasize that, due to the presence of the regularizer term in (1.1), this step constructs a map that is ideally not far from a physical solution.

Subsequently, in the corrector part, the strain energy is minimized while the similarity obtained earlier remains *the same* by applying the distance function. As shall be discussed, the resulting solution corresponds to an equivalent Dirichlet boundary value problem where the displacement boundary conditions are dictated based on the predictor map.

Moreover, to guarantee the invertibility of the (predictor) corrector map, we employ neural ordinary differential equations (NODEs) and define the transformation in terms of its (pseudo) velocity, making the method conceptually in line with diffeomorphism algorithms (Miller et al., 2002). We note that the registration problem is ill-posed in its nature in the sense that the map aligning the target and initial images is not unique. Hence, the resulting solution of the present predictor-corrector algorithm represents one of the infinitely many admissible transformations for the registration problem. However, in contrast to previous methods where a multi-objective optimization was only adapted, the main features of the present method are: 1) the method is an integral variant of PINNs, and consequently, it avoids calculation of second spatial derivatives, 2) the predictor part minimizes the mismatch loss in a better fashion because it does not need to solve the physics problem concurrently, 3) the corrector part solves a physics problem without losing the registration, allowing for accurate modeling of complex biophysics e.g. growth and remodeling.

**Fig. 1:**
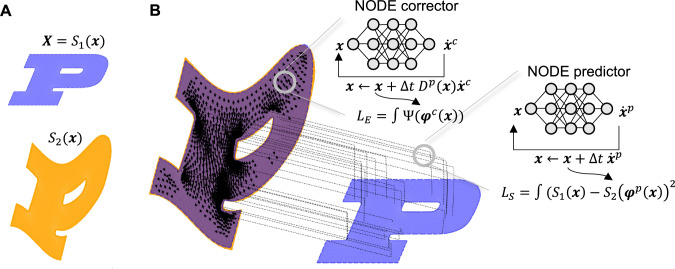
Method overview. A) Source and target images. B) The method consists of two steps. In a predictor step, a neural ordinary differential equation (NODE) is used to map the source to the target by minimizing a mismatch loss with a regularization term. The NODE guarantees an invertible map provided a small enough time step is used. In a corrector step, the map found by the predictor is enforced through distance functions, and the loss is that of a physics problem, e.g., minimization of total elastic energy

As shown in Pawar et al. (2022), the solution of a multi-objective function converges to the one that balances the mismatch and the regularizer losses, which is a function of the hyper-parameter $\beta$. This interplay means that neither problem is solved adequately. Hence, here in the predictor part, we decrease the hyper-parameter $\beta$ as much as possible so that we can obtain a diffeomorphic map that minimizes the mismatch loss in a more accurate manner. The resulting transformation from the corrector systematically satisfies the equilibrium equations of continuum mechanics, including the growth equations, and thus may represent underlying physics in a better fashion. Furthermore, many biomedical applications require a multi-physics coupling, e.g., growth and remodeling (Ambrosi et al., 2011). We also introduce such a coupling in this manuscript to capture embryo development and brain atrophy. Fig 1 shows the main steps of the new method.

The remainder of the paper is organized as follows: firstly, we enumerate the main equations of solid mechanics. Then, the concept of NODE combined with the forward Euler method is briefly reviewed, and the underlying reason to consider this architecture is explained. Next, the theory of the predictor-corrector algorithm is explained, where the incorporation of the growth problem is omitted for clarity. Specifically, it is shown how the new solution procedure directly embodies the energy (variational) method, which is the foundation of any FEM, and accordingly, the new method represents a physically meaningful deformation defined as an invertible map between source and target images that systematically fulfills an equilibrium equation. Subsequently, the theory that incorporates growth is developed, where a first-order evolutionary ODE is introduced to model the addition of mass. The numerical results for two- and three-dimensional models with rather simple geometry are defined to highlight features of the method and registration problem. Subsequently, three biomedical practical problems with the presence of growth are investigated: 1) growth in the embryo of zebrafish tail, 2) shrinkage of the brain with aging, and 3) fetal brain development. Finally, an ablation study is performed to better understand capability of the method.

## Background

### Fundamental Equations

Let us assume the initial and target images define domains containing, respectively, undeformed and deformed configurations of an elastic continuum, denoted by $\mathscr {B}^I$ and $\mathscr {B}^T$. We also show the whole domain of integral with $\Omega$. Then the equilibrium equations read as:

2.1 $$\begin{aligned} \begin{aligned} {\nabla _{\textbf{X}}}\cdot \,{\textbf{P}} + {\rho _0}{\textbf{b}} = 0,\,\,\,\, {\textbf{P}}{{\textbf{F}}^T} ={\textbf{F}}{{\textbf{P}}^T}\,\,\,on\,\,\,{\mathscr {B}^I}, \end{aligned} \end{aligned}$$

in which, ${\nabla _{\textbf{X}}}\cdot$ is the divergence operator with respect to material coordinates and $\textbf{P}$, $\textbf{F}=\frac{\partial \mathbf{{x}}}{\partial \mathbf{{X}}}$, and $\rho _0$ are the first Piola Kirchhoff stress, deformation gradient, and reference density, respectively. Moreover, by assuming hyper-elasticity, we can write:

2.2 $$\begin{aligned} \begin{aligned}&{\textbf{S}} = \;2{\rho _0}\frac{\partial \psi ({\textbf{C}})}{\partial \textbf{C}} \,\,\,\,\,\, \text {on} \,\,{\mathscr {B}^I},\,\,{\textbf{S}} = {{\textbf{F}}^{ - T}}{\textbf{P}},\,\,{\textbf{C}} = {{\textbf{F}}^T}{\textbf{F}},\ \end{aligned} \end{aligned}$$

with $\psi$, $\textbf{C}$, and $\textbf{S}$ are a convex potential function, the Cauchy strain tensor, and the second Piola Kirchhoff stress, respectively. The above equilibrium and constitutive equations define a mixed boundary value problem by considering the following prescribed boundary conditions:

2.3 $$\begin{aligned} \begin{aligned}&\textbf{u} ={{{\textbf{G}}}}(\mathbf{{X}})&\,\,\,\,\,\,\,\,\text {on}\,\,\,\, \partial \mathscr {B}^I_{{u}},\\&\textbf{PN}={{\textbf{H}}}(\mathbf{{X}})&\,\,\,\,\,\, \text {on}\,\,\,\partial \mathscr {B}^I_{{t}}, \end{aligned} \end{aligned}$$

where $\partial \mathscr {B}^I_{{u}}$ and $\partial \mathscr {B}^I_{{t}}$ are disjoint and complementary sets of boundary points with prescribed displacement and traction boundary conditions ${{\textbf{G}}}$ and ${{\textbf{H}}}$, and $\textbf{N}$ is the unit normal vector on the boundary of $\partial \mathscr {B}^I$.

A systematic approach to solve the above mixed-boundary value problem is to satisfy the localized (partial differential) equations weakly through an integral sense. Indeed, the solution of the problem is the stationary point of the following integral form:

2.4 $$\begin{aligned} \begin{aligned} \mathop {\arg \min }\limits _{\textbf{u}} \,\,\Pi ({\textbf{u}})&= \int \limits _{{\mathscr {B}^I}} {\psi ({\textbf{C}})d\Omega } - \int \limits _{{\mathscr {B}^I}} {{\rho _0}{\textbf{b}}.{\textbf{u}}d\Omega } - \int \limits _{\partial \mathscr {B}_t^I} {{\textbf{PN}}.\,{\textbf{u}}d\Gamma } ,\\&{\textbf{u}} = {\textbf{x}} - {\textbf{X}}, \\&\textbf{u} ={{{\textbf{G}}}}(\mathbf{{X}})\,\,\,\,\,\,\,\text {on}\,\,\,\, \partial \mathscr {B}^I_{{u}}. \end{aligned} \end{aligned}$$

Now, in the case of the purely Dirichlet boundary condition and absence of body force, the above equation reduces to:

2.5 $$\begin{aligned} \begin{aligned} \mathop {\arg \min }\limits _{\textbf{u}} \,\,\Pi ({\textbf{u}})&= \int \limits _{{\mathscr {B}^I}} {\psi ({\textbf{C}})d\Omega }, \end{aligned} \end{aligned}$$

which is central to our registration method. In the FEM, the satisfaction of the above equations is achieved uniformly in the domain by discretization and approximately vanishing the first variation of the above functional, while here, we define (2.5) as the loss function and directly process the minimization of the total potential energy, which is equivalent.

### NODE Architecture

In continuum mechanics, admissible solutions are smooth and invertible. The invertibility of the deformation field leads to $J=\det (\textbf{F})>0$. In the classical FEM, the displacement of a point is interpolated based on the displacement of surrounding nodal points. Hence, the above-mentioned non-negativity condition on Jacobian is identically fulfilled as long as the interpolation is regular. In contrast, in neural network architectures, a stack of nonlinear and linear transformations results in a map that is not necessarily injective.

One significant property of invertible maps is that they are closed under composition. As a result, it is possible to build complex invertible transformations by composing invertible building blocks, establishing a new family of invertible maps that are called flow methods (Papamakarios et al., 2021), which found applications in generative models. NICE (Dinh et al., 2014), and real NVP (Dinh et al., 2016) are notable examples of discrete flow algorithms that have a predefined structure in each building block. The invertible residual networks (Behrmann et al., 2019; Chen et al., 2019)is another example that is defined as

2.6 $$\begin{aligned} \begin{aligned} {\textbf{x}} = {\textbf{X}} + NN({\textbf{X}};{\textbf{W}},{\textbf{b}}), \end{aligned} \end{aligned}$$

where the function is a neural network with weights ${\textbf{W}}$ and biases ${\textbf{b}}$. As mentioned in Behrmann et al. (2019), Chen et al. (2019), a sufficient condition for (2.6) to be invertible is when *NN* is contractive in the sense of:

2.7 $$\begin{aligned} \begin{aligned} {d_x}(NN({\mathbf{{X}}_2}),NN({\mathbf{{X}}_1})) \leqslant K {d_X} ({\mathbf{{X}}_2},{\mathbf{{X}}_1}),\,\,\,\,\, K<1, \end{aligned} \end{aligned}$$

where *K* is a constant independent of $X_1$ and $X_2$, and $d_x$ and $d_X$ are metrics defined in space $\textbf{x}$ and $\textbf{X}$, respectively. Hence, one can define a stack of residual networks with a parameter $\Delta t$:

2.8 $$\begin{aligned} \begin{aligned} {{\textbf{x}}^{(i + 1)}} = {{\textbf{x}}^{(i)}} + \Delta t\,NN({{\textbf{x}}^{(i)}}) \end{aligned} \end{aligned}$$

in which $\Delta t$ is small enough such that $\Delta t\,NN({{\textbf{x}}^{(i)}}), i=1,...,n$ is contractive.

Apparently, the above formulation is the forward Euler time integration of the following ODE:

2.9 $$\begin{aligned} \begin{aligned} \frac{{d{\textbf{x}}}}{{dt}} = NN({\textbf{x}};{\textbf{w}},{\textbf{b}}), \end{aligned} \end{aligned}$$

which is equivalent to the concept of Neural ODE (Chen et al., 2018). Additionally, (2.7) is analogous to diffeomorphism algorithms (Christensen et al., 1994, 1996). Hence, in the present method, to obtain invertible transformation, we employ (2.8) and define $\Delta t$ such that we guarantee (2.7).

## Method

### Predictor

In the predictor part, the main focus is to attain a map that ideally aligns two images. To this end, we follow the classical formulation of registration, where the transformation is obtained from the minimization of a loss function consisting of similarity and regularization terms. Given two (binary) images discretized in pixels denoted by *T* and *I*, following (Pawar et al., 2022), we define two functions ${S_1}({\textbf{X}})$ and ${S_2}({\textbf{x}})$ as follows:

3.1 $$\begin{aligned} \begin{aligned}&{S_1}({\textbf{X}},\mathscr {B}^I) = \sum \limits _i {{c_i}{\xi _i}({\textbf{X}})} ,\,\,\,{c_i} = \left\{ \begin{gathered} 1,\,\,\,\,\,\,\,\,{{\textbf{X}}_i} \in {\mathscr {B}^I} \\ 0,\,\,\,\,\,\,\,{\text {otherwise}} \\ \end{gathered} \right. ,\\&{S_2}({\textbf{x}},\mathscr {B}^T) = \sum \limits _i {{d_i}{\xi _i}({\textbf{x}})} ,\,\,\,{d_i} = \left\{ \begin{gathered} 1,\,\,\,\,\,\,\,\,{{\textbf{x}}_i} \in {\mathscr {B}^T} \\ 0,\,\,\,\,\,\,\,{\text {otherwise}} \\ \end{gathered} \right. , \end{aligned} \end{aligned}$$

where ${{\xi _i}}$ are (linear Lagrange ) interpolating functions, and ${{\textbf{x}}_i}$ and ${{\textbf{X}}_i}$ are nodal coordinates corresponding to $d_i$ and $c_i$, respectively. Then, the predictor map is obtained from the minimization of the following loss function:

3.2 $$\begin{aligned} \begin{aligned}&\mathop {\arg \min }\limits _{{{\textbf{w}}_p},{{\textbf{b}}_p}{\hspace{1.0pt}} } \left\{ \int \limits _\Omega {{{\left( {{S_1}({\textbf{X}},{\mathscr {B}^I}) - {S_2}\left( {{\textbf{x}}_p^{({n_p})}\left( {{{\textbf{w}}_p}, {{\textbf{b}}_p}} \right) ,{\mathscr {B}^T}} \right) } \right) }^2}d\Omega } \right. \\&\quad \left. + \beta \int \limits _\Omega {R\left( {{S_1}({\textbf{X}},{\mathscr {B}^I})\left( {{\textbf{x}}_p^{({n_p})}\left( {{{\textbf{w}}_p}, {{\textbf{b}}_p}} \right) - {\textbf{X}}} \right) } \right) d\Omega } \right\} , \end{aligned} \end{aligned}$$

where

3.3 $$\begin{aligned} \begin{aligned}&{{\textbf{x}}_p^{(i + 1)}} = {{\textbf{x}}_p^{(i)}} + \Delta \tau \, {\textbf{NN}}({{\textbf{x}}}_p^{(i)};{\textbf{w}}_p,{\textbf{b}}_p),\,\,\,\,\,\\&\quad i=0,...,n_p-1,\,\, \Delta \tau =\frac{1}{n_p},\\&\quad {{\textbf{x}}_p^{(0)}} = {\textbf{X}}, \end{aligned} \end{aligned}$$

in which ${\textbf{w}}_p$ and ${\textbf{b}}_p$ denote parameters of the neural network for predictor transformation, which are determined by minimization of the functional (3.2), *R* is a regularization term that typically depends on $\textbf{F}$, and $n_p$ is defined to guarantee invertibility of the resulting transformation. In this study, for synthetic and biological examples, we utilize the strain energy density function as the regularization term, i.e., $R=\psi (\textbf{F})$. However, in the ablation study part, we show that the method is applicable when the regularizing term is different than the strain energy potential. This fact implies that the method can successfully adapt admissible geometrical registration data obtained from other methods and attach physics by converting the predictor deformation into a distance function and applying the corrector part with this constraint. Different strain energy models could be used, but we opt for a neo-Hookean model or the St Venant Kirchhoff model. Additionally, in (3.2), $\beta$ is a hyperparameter that may vary from problem to problem. Nevertheless, in the new method, a small value for $\beta$ is considered such that the predictor deformation field produces an admissible transformation in the sense of non-vanishing Jacobians while a satisfactory registration can be achieved. This is in contrast to classical methods, where the registration is entirely carried out by (3.2), and hence, the choice of the hyperparameter is crucial to find a good map. For us, as long as the predictor map is injective, regularity in the sense of equilibrium of large deformation elasticity is systematically validated and enforced in the corrector step.

### Corrector

By employing (3.2), one can *optimally* obtain a smooth transformation that maps the boundary points of the initial image into the boundary points of the target image. However, the aforementioned transformation does not necessarily satisfy the equilibrium equations for interior points, even if the regularizing term is selected as the strain potential energy. Thus, the physics of the problem needs to be validated and enforced as an additional step. In doing so, we present an approach that, similar to FEMs, seeks to obtain a stationary point of total potential energy while maintaining the alignment of the predictor.

In doing so, having obtained the *predictor* solution from (3.2), we define the following Dirichlet boundary value problem:

3.4 $$\begin{aligned} \begin{aligned}&{\nabla _{\textbf{X}}} \cdot {\textbf{P}} = {\textbf{0}},\\&{\textbf{u}}({\textbf{X}}) = {{\textbf{x}}_p^{(n_p)}} - {\textbf{X}}\,\,\,\,\,\,\,{\text {on}}\,\,{\mathscr {B}^I} \end{aligned} \end{aligned}$$

Then, to satisfy (3.4)$_1$, we alternatively minimize the potential energy (2.5):

3.5 $$\begin{aligned} \begin{aligned} \mathop {\arg \min }\limits _{{{\textbf{w}}_c}, {{\textbf{b}}_c}} \Pi = \int \limits _\Omega {\psi \left( {{S_1}({\textbf{X}},{\mathscr {B}^I})\left( {{\textbf{x}}_c^{({n_c})}\left( {{{\textbf{w}}_c}, {{\textbf{b}}_c}} \right) - {\textbf{X}}} \right) } \right) d\Omega } , \end{aligned} \nonumber \\ \end{aligned}$$

where we consider the following form for the correction:

3.6 $$\begin{aligned} \begin{aligned}&{\textbf{x}}_c^{(i + 1)} = {\textbf{x}}_c^{(i)} + \Delta t{D_p}({\textbf{x}}_c^{(i)};{\textbf{x}}_{{\mathscr {B}^I}}^{p(n_p)})\,{\textbf{NN}}({\textbf{x}}_c^{(i)};{{\textbf{w}}_c},{{\textbf{b}}_c}),\,\,\,\\&\quad i = 0,...,{n_c-1},\,\,\,\Delta t = \frac{1}{{{n_c}}},\\&\quad {{\textbf{x}}_c^{(0)}} = {{\textbf{x}}_c^{({n_p})}}. \end{aligned} \end{aligned}$$

$D_p$, in (3.6), can be any function that fixes the boundary condition imposed by the predictor solution while the interior points are transformed to minimize (3.2). A suitable choice, in this regard, is the distance function:

3.7 $$\begin{aligned} \begin{aligned} {D_p}({\textbf{x}};\,{\textbf{x}}_{{\mathscr {B}^I}}^{^{p(n)}}) = \min (\left\| {{\textbf{x}} - {\textbf{x}}_{{\mathscr {B}^I}}^{{p(n)}}} \right\| ) \end{aligned} \end{aligned}$$

in which || || indicates the Euclidean norm, and ${\textbf{x}}_{{\mathscr {B}^I}}^{^{p(n)}}$ denotes the set of transformed boundary points by predictor map. While there are several methods available to approximate the above distance functions (see, for example, Berg and Nyström (2018) and Sukumar and Srivastava (2022)), we shall directly utilize (3.7) since in the numerical part we deal with complex 3d geometry such as brains. Thus, in the numerical implementation, we approximate the distance function based on a finite representation of ${\textbf{x}}_{{\mathscr {B}^I}}^{^{p(n)}}$. Note that there is no need to use the same potential for the predictor and corrector steps. For the corrector step, one could adopt more complex material behavior specific to a particular soft tissues, e.g. brain (Mihai et al., 2015), skin Limbert (2014), or myocardium Holzapfel and Ogden (2009), to name a few examples. Multi-physics coupling can also be introduced. For instance, in the following section, we present the formulation for finite volume growth biophysics as part of the corrector (Ambrosi et al., 2011).

###  Finite Growth Biophysics

All living biological tissues show behavior beyond large deformation elasticity, including coupled electrophysiology (Göktepe & Kuhl, 2009), reaction-diffusion (Tepole, 2017), and ability to grow and remodel (Ambrosi et al., 2011). Here, we tackle that last one by introducing the multiplicative split of the deformation gradient

3.8 $$\begin{aligned} \begin{aligned} {\textbf{F}} = {{\textbf{F}}^e}{{\textbf{F}}^g}, \end{aligned} \end{aligned}$$

where ${{\textbf{F}}^e}$ and ${{\textbf{F}}^g}$ indicate elastic and inelastic or growth deformation, respectively. The first consequence of such a split is that the energy in the corrector is assumed to depend only on the elastic deformation $\psi ({{\textbf{F}}^e})$. In FEM implementations of growth, or if the strong form was desired, one should be careful with the computation of the stress with the appropriate pull-back or push-forward operations from the intermediate state (Himpel et al., 2005). We do not encounter such a problem here because the energy is minimized directly. In addition to $\psi ({{\textbf{F}}^e})$, the second change to the corrector is the introduction of a rate equation with an initial condition for the growth tensor $\textbf{F}^g$.

Assuming isotropic homogeneous growth:

3.9 $$\begin{aligned} \begin{aligned} {{\textbf{F}}^g} = {\theta ^g}{\textbf{I}}, \end{aligned} \end{aligned}$$

where the scalar $\theta ^g$ captures the addition of volume at constant reference density $\rho _0$ (Himpel et al., 2005). Other options for growth tensor are to model fiber or area growth (Eskandari & Kuhl, 2015). In many cases, the kinetic equation for growth update depends on the elastic deformation $\dot{\theta }^g(\textbf{F}^e)$ (Eskandari & Kuhl, 2015). However, particularly for brain development or atrophy shown here, the growth rate can be assumed to be morphogenetic or dependent on the concentration of certain chemicals that drive growth or atrophy, and the dependence on $\textbf{F}^e$ can be ignored (Weickenmeier et al., 2018; Wang et al., 2021). Assuming exponential growth independent of deformation and initial condition $\theta ^g(0)=1$, one obtains

3.10 $$\begin{aligned} \begin{aligned} {\theta ^g}(t) = \exp ( \kappa t). \end{aligned} \end{aligned}$$

where $\kappa$ is the growth rate parameter. Hence, for the predictor part in the biological growth examples, we solve the same problem as before (3.2), while in the corrector, we solve the following problem:

3.11 $$\begin{aligned} \begin{aligned} \mathop {\arg \min }\limits _{{{\textbf{w}}_c},{\hspace{1.0pt}} {{\textbf{b}}_c},\kappa } \Pi = \int \limits _\Omega {\psi \left( {{S_1}\left( {{\textbf{X}},{\mathscr {B}^I}} \right) \left( \textbf{F}^e \right) ,\kappa } \right) d\Omega } \end{aligned} \end{aligned}$$

where it can be seen that we optimize for the deformation as well as for the growth $\kappa$ such that elastic energy is minimized.

### Sequential Approach Coupled With Growth

In this part, we explain the method when a stack of images as a function of time is considered. In this regard, we assume that changes through time are slow enough to neglect the inertial force in our analysis. Considering the algorithm mentioned earlier for single registration, there are several possibilities for carrying out the registration for sequential image data. In this paper, given an initial image and sequence of images, which are indicated by $I_0$ and $I_j, j=1,...,n_s-1$, with the pertinent predictor shown by ${\textbf{x}}_{p(j)}^{({n_p})},j = 1,...,{n_s-1},{\textbf{x}}_{p(0)}^{({n_p})} = {\textbf{X}}$, we define the forward composition function:

3.12 $$\begin{aligned} \begin{aligned} {\textbf{x}}_{p(0 \rightarrow j)}^{({n_p})} \equiv {\left\{ \begin{array}{ll} & {\textbf{X}},\,\,\,\,j = 0\\ & {\textbf{x}}_{p(j)}^{({n_p})} \circ {\textbf{x}}_{p(j - 1)}^{({n_p})} \circ ... \circ {\textbf{x}}_{p(1)}^{({n_p})}({\textbf{X}}),\,\,\,{\text {otherwise}}\\ \end{array}\right. } \end{aligned} \nonumber \\ \end{aligned}$$

Analogously, we define the backward composition function, ${\textbf{X}}_{p(k \rightarrow 0)}^{({n_p})}$, as follows

3.13 $$\begin{aligned} \begin{aligned} {\textbf{X}}_{p(j \rightarrow 0)}^{({n_p})} \equiv {\left\{ \begin{array}{ll} & {\textbf{X}},\,\,\,\,j = 0\\ & {\textbf{X}}_{p(1)}^{({n_p})} \circ {\textbf{X}}_{p(2)}^{({n_p})} \circ ... \circ {\textbf{X}}_{p(j)}^{({n_p})}({\textbf{X}}),\,\,\, \,\,\,\,\,{\text {otherwise}}\\ \end{array}\right. } \end{aligned} \end{aligned}$$

in which ${\textbf{X}}_{p(k)}^{({n_p})}({\textbf{X}})$ denotes the inverse map of the $k^{th}$ step, which can be approximated from the following recursive equation by setting $i=n_p-1$:

3.14 $$\begin{aligned} \begin{aligned}&{\tilde{\textbf{X}}}_p^{(0)} = {\textbf{ X}}\\&\quad {\tilde{\textbf{X}}}_p^{(i + 1)} = {\tilde{\textbf{X}}}_p^{(i)} - \Delta \tau NN({\tilde{\textbf{X}}}_p^{(i)};{{\textbf{w}}_{p(k)}}, {{\textbf{b}}_{p(k)}});\, \,\,\, \\&\quad i = 0,...,{n_p} - 1;\,\,\,\Delta \tau = \frac{1}{{{n_p}}}. \end{aligned} \end{aligned}$$

Then, the predictor part may be constructed in the sequential format of (3.2):

3.15 $$\begin{aligned} \begin{aligned}&\mathop {\arg \min }\limits _{{{\textbf{w}}_{p(j)}},{{\textbf{b}}_{p(j)}}} \\&\quad = \left\{ \int \limits _\Omega {{{\left( {{S_1}\left( {{\textbf{X}}_{p(j-1 \rightarrow 0)}^{({n_p})},{\mathscr {B}^{{I_0}}}} \right) - {S_2}\left( {{\textbf{x}}_{p(j)}^{({n_p})},{\mathscr {B}^{{I_j}}}} \right) } \right) }^2}d\Omega }\right. \\&\quad \left. + \beta \int \limits _\Omega {R({S_1}\left( {{\textbf{X}},{\mathscr {B}^{{I_0}}}} \right) \left( {{\textbf{x}}_{p(0 \rightarrow j)}^{({n_p})} - {\textbf{X}}} \right) )d\Omega } \right\} ,\\&\quad {{\textbf{x}}_{p(0)}} = {\textbf{X}},\,\, j = 1,...,{n_s-1}, \end{aligned} \end{aligned}$$

The first term of the above integral can be simplified, provided that an acceptable registration is performed. That is, assuming a good registration, one can use the following approximation in (3.15):

3.16 $$\begin{aligned} \begin{aligned} {S_1}\left( {{\textbf{X}}_{p(j-1 \rightarrow 0)}^{({n_p})},{\mathscr {B}^{{I_0}}}} \right) \approx {S_1}\left( {{\textbf{X}},{\mathscr {B}^{{I_{j - 1}}}}} \right) \,\,\, j = 1,...,{n_s-1}, \end{aligned}\nonumber \\ \end{aligned}$$

leading to the following minimization:

3.17 $$\begin{aligned} \begin{aligned}&\mathop {\arg \min }\limits _{{{\textbf{w}}_{p(j)}},{{\textbf{b}}_{p(j)}}} \\&\quad = \left\{ \int \limits _\Omega {{{\left( {{S_1}\left( {{\textbf{X}},{\mathscr {B}^{{I_{j - 1}}}}} \right) - {S_2}\left( {{\textbf{x}}_{p(j)}^{({n_p})},{\mathscr {B}^{{I_j}}}} \right) } \right) }^2}d\Omega }\right. \\&\quad \left. + \beta \int \limits _\Omega {R({S_1}\left( {{\textbf{X}},{\mathscr {B}^{{I_0}}}} \right) \left( {{\textbf{x}}_{p(0 \rightarrow j)}^{({n_p})} - {\textbf{X}}} \right) )d\Omega } \right\} ,\\&{{\textbf{x}}_{p(0)}} = {\textbf{X}},\,\, j = 1,...,{n_s-1}. \end{aligned} \end{aligned}$$

In this paper, we used (3.17) to obtain the predictors. Subsequently, for the corrector part, we define:

3.18 $$\begin{aligned} \begin{aligned} {\textbf{x}}_{pc(k)}^{({n_p,n_c})} \equiv {\textbf{x}}_{c(k)}^{({n_c})} \circ {\textbf{x}}_{p(k)}^{({n_p})},\,\,\, k=1,...,n_s-1. \end{aligned} \end{aligned}$$

Next, we modify (3.12) as:

3.19 $$\begin{aligned} \begin{aligned} {\textbf{x}}_{pc(0 \rightarrow j)}^{({n_p},{n_c})} \equiv {\textbf{x}}_{pc(j)}^{({n_p},{n_c})} \circ {\textbf{x}}_{pc(j - 1)}^{({n_p},{n_c})} \circ ... \circ {\textbf{x}}_{pc(1)}^{({n_p},{n_c})},\,\,\, j=1,...,n_s-1. \end{aligned}\nonumber \\ \end{aligned}$$

The corrector solution for each pair of images is then obtained consecutively by using the following:

3.20 $$\begin{aligned} \begin{aligned} \mathop {\arg \min }\limits _{{{\textbf{w}}_{c(j)}},\,\,{{\textbf{b}}_{c(j)}},{\kappa _{(j)}}} \Pi = \int \limits _\Omega {\psi \left( {{S_1}\left( {{\textbf{X}},{\mathscr {B}^{{I_0}}}} \right) \left( {{\textbf{x}}_{pc(0 \rightarrow j)}^{({n_p},{n_c})} - {\textbf{X}}} \right) } \right) d\Omega } , \end{aligned}\nonumber \\ \end{aligned}$$

such that the minimization of the energy in the corrector steps always keeps track of the deformation with respect to the initial configuration even though the predictor and corrector steps are done sequentially.

## Numerical Results

To show the potential of the method, we provided several examples, including synthetic benchmarks, followed by three practical applications. In synthetic and biological examples, for constitutive equations, we consider either Saint Venant-Kirchhoff material with the following strain energy potential:

4.1 $$\begin{aligned} \begin{aligned} \psi ({\textbf{C}}) = \frac{\lambda }{2}\left( {{\text {tr}}{\textbf{E}}} \right) + \mu \,{\text {tr}}\left( {{{\textbf{E}}^2}} \right) ,\,\,{\textbf{E}} = \left( {\frac{{{\textbf{C}} - {\textbf{I}}}}{2}} \right) , \end{aligned} \end{aligned}$$

or neo-Hookean material:

4.2 $$\begin{aligned} \begin{aligned} \psi ({\textbf{C}}) = \frac{\mu }{2}({\text {tr}}{\textbf{C}} - 3) - \mu \log \left( J \right) + \frac{\lambda }{2}{\left( {\log \left( J \right) } \right) ^2}, \end{aligned} \end{aligned}$$

where for simplicity, we assume $\mu =\lambda =1 Pa$ in all examples. For the ablation study, we consider the following strain potential function:

4.3 $$\begin{aligned} \begin{aligned}&\psi ({\textbf{C}}) = 3{\left( {J - 1} \right) ^2} + \left( {{I_1} - 3} \right) + (I_2-3)/12+{\left( {{I_1} - 3} \right) ^2}\\&{I_1} = {\text {tr}}{\textbf{C}},\,\,{I_2} = \frac{1}{2}\left( {{{\left( {{\text {tr}}{\textbf{C}}} \right) }^2} - {\text {tr}}{{\textbf{C}}^2}} \right) ,\,J = \sqrt{\det {\textbf{C}}} . \end{aligned} \end{aligned}$$

During the learning process, we employed the Adam optimizer with a learning rate of 0.00005. The choice of $\beta$ varied depending on the problem, with specific values to be mentioned accordingly. Additionally, the architecture of the neural network used in the NODE consists of three hidden layers, each containing 40 neurons for 2d and 60 neurons for 3d problems, which is in the range taken for similar problems in the literature (see the neural network architectures taken in Sukumar and Srivastava (2022), Bai et al. (2023)). Furthermore, we take 15 steps in the NODE, i.e., $\Delta t= \Delta \tau =1/15$ for both predictor and corrector. We evaluated the integrals by discretizing the domain into $200\times 200$ elements for 2d problems and $45\times 45\times 45$ elements for 3d problems. In each direction, we considered two integration points for every element, i.e., four points for 2d elements and eight points for 3d elements. This process involved using a mini-batch of 4000 uniformly randomly distributed elements in the predictor part for each epoch. In the corrector part, we utilized mini-batches of 2000 and 1500 uniformly randomly distributed elements in each epoch for 2d and 3d examples, respectively.

### Synthetic Examples

For synthetic examples, we explore non-uniform deformations, including extension, shearing, and bending modes of deformation, applied to a multiple-connected plate. Furthermore, we compare the results obtained when using analytical expressions for $S_1$ and $S_2$ against discretization in pixels. We further investigate the impact of using binary data versus RGB image data with 256-bit information per channel, thereby providing more detailed information than binary data.

**Fig. 2:**
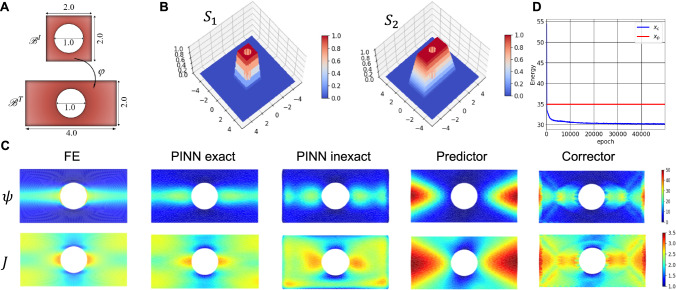
Comparison of registration, physics-informed neural networks, and finite element analysis: A) extension of a plate with the whole; undeformed and deformed configurations. B) 3d views of analytic $S_1$ and $S_2$ functions. C) strain energy density and Jacobian fields obtained from different methods, including finite element method, integral form of the PINN when the Dirichlet boundary conditions are imposed exactly, integral form of PINN when the boundary conditions are imposed as penalty terms, predictor part of the registration, and corrector solution

For the first example, we analyze the extension of a Saint Venant-Kirchhoff plate with a hole. The displacement boundary conditions are as follows:

4.4 $$\begin{aligned} \begin{aligned}&{u_1}( - 1,{X_2}) = - 1,{u_1}(1,{X_2}) = 1,\\&{u_2}({X_1}, - 1) = {u_2}({X_1},1) = 0,\\&{u_1}({X_1},{X_2}) = {u_2}({X_1},{X_2}) = 0,\,\,\,X_1^2 + X_2^2 = 0.25 \end{aligned} \end{aligned}$$

and traction-free boundary conditions are assumed for the Neumann boundary conditions. The FEM results are used as a reference solution. For the *PINN inexact* approach, the hyper-parameter for displacement boundary conditions has been set to one, while 0.01 was selected as the hyper-parameter pertinent to strain energy. Additionally, in the predictor part of the new approach, $\beta = 1/6000$ was considered. Results pertinent to the energy and Jacobian have been plotted in Fig 2-C labeled with *FE*. Fig2-C, *PINN exact* column, are the solutions obtained from a PINN-like by minimizing the strain energy with the imposition of exact boundary conditions. In contrast to classical PINNs (Raissi et al., 2019), or variational PINNs (Kharazmi et al., 2019), we minimize the energy and not the residual of the weak form or the strong form. As can be seen, there is a good agreement between the energy form of the PINN with the exact imposition of boundary conditions and the FEM solution. The results of the integral energy form of the PINN with enforcing boundary conditions as a penalty term have been depicted in Fig2-C, labeled with *PINN inexact*. In comparison to the FEM results, there are some discrepancies in the results of PINN with penalty terms. In particular, the displacement boundary conditions have not been exactly satisfied over the boundary of the hole, while the resulting energy and Jacobian fields have some disagreement with those corresponding to FEM. This discrepancy can be attributed to the fact that PINN when boundary conditions are enforced with penalty terms, is a multi-objective optimization, and thus, the resulting solution generally satisfies both objects to some extent.

We next analyze the registration problem, where we do not have complete information regarding the boundary conditions. We employ an analytical format for $S_1$ and $S_2$ (see Fig. 2-B). The results for the predictor part have been reported in Fig. 2-C, labeled with *Predictor*. As can be seen, for the registration case, incomplete information regarding the boundary condition and small value of the regularization $\beta$ leads to discrepancies in the energy and Jacobian fields with respect to FEM, but the predictor achieves accurate registration with a smooth invertible map. Subsequently, the results after applying the corrector part have been illustrated in Fig. 2-C labeled with *Corrector*. The difference between the results of the corrector and predictor is shown. The path of minimization of the total potential energy in the corrector part has also been shown in Fig. 2-D. It is worth noting that, as the name suggests, the corrector part is designed to guarantee the minimization of the potential energy corresponding to the Dirichlet boundary value problem specified by the predictor. In addition, we quantified how the corrector maintains the quality of registration by calculating the Dice index across the entire domain. In segmentation analysis, the quality of registration is typically measured using the Dice index, which is defined as follows:

4.5 $$\begin{aligned} \begin{aligned} {\text {Dice(S}}_2^k,S_1^k \circ {{\mathbf {\varphi }}^{ - 1}}) = 2\frac{{\left| {{\text {S}}_2^k \cap \left( {S_1^k \circ {{\mathbf {\varphi }}^{ - 1}}} \right) } \right| }}{{\left| {{\text {S}}_2^k} \right| + \left| {S_1^k \circ {{\mathbf {\varphi }}^{ - 1}}} \right| }}, \end{aligned} \end{aligned}$$

where superscript k denotes the $k^{th}$ region of interest and $S_1^k \circ {{\mathbf {\varphi }}^{ - 1}}$stands for the composition of S1 with the inverse of transformation. This function basically constructs the predicted values of $S_2$ for direct comparison to the ground truth $S_2$ by mapping the $S_1$ intensities to the $S_2$ coordinate grid using the NODE map. In this example, the Dice index for predictor and corrector parts are *0.95* and *0.948*, indicating good maintenance of the registration in the corrector part.

As explained earlier, the existence of the corrector part in the method is essential. The multi-objective format of the predictor does not necessarily lead to a physically meaningful solution. Especially if a relatively low regularization $\beta$ is required to achieve a good alignment between initial and target images, particularly in large deformations, the transformation can be far from equilibrium. Another fact that should be noticed is that even if the predictor produces an excellent alignment, it does not necessarily lead to the same boundary value problem with similar Dirichlet boundary conditions defined in FEM. In fact, there are infinitely many predictor solutions that minimize the misalignment but lead to a different Dirichlet problem. This is not a limitation of the method, though, but rather a difference between the nature of registration and classical well-defined boundary value problems in physics where complete boundary information is available. Thus, the corrector solution in Fig. 2-C solves a Dirichlet problem, just not the same problem as the one solved with FEM or PINNs.

As the next example, we consider the shear deformation of the neo-Hookean plate with the hole while the hole is under contraction. In this regard, the following displacement boundary conditions have been imposed:

4.6 $$\begin{aligned} \begin{aligned}&{u_1}( - 1,{X_2}) = {u_1}(1,{X_2}) = 0.25\left( {{X_2} + 1} \right) ,\\&{u_2}( - 1,{X_2}) = {u_2}(1,{X_2}) = 0,\\&{u_2}({X_1}, - 1) = {u_2}({X_1},1) = 0,\\&{u_r}({X_1},{X_2}) = 0.2,\,\,{u_\theta }({X_1},{X_2}) = 0,\,\,\,X_1^2 + X_2^2 = 0.25,\,\,\, \end{aligned} \end{aligned}$$

and the traction boundary conditions are vanishing.

We utilize this example to compare the performance of the general neural network with the NODE in generating an admissible deformation field in continuum mechanics. For the NODE, we utilized (3.2) with $\beta =1/6000$. We do not consider the corrector part in this example.

**Fig. 3:**
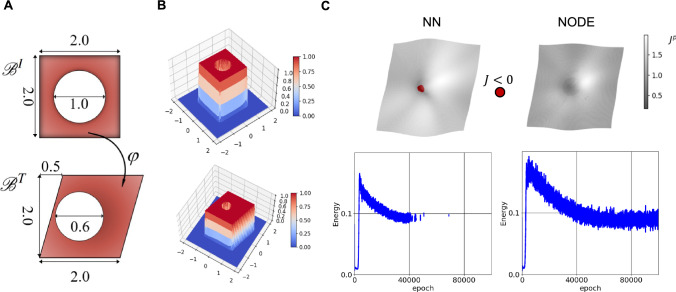
The performance of the resulting deformation field was evaluated when the displacement field is approximated using neural networks, compared to when the (pseudo) velocity is constructed based on neural networks (Neural ODE registration) A) extensive shear deformation of the neo-Hookean plate with the whole; undeformed and deformed configurations. B) 3d views of analytic $S_1$ and $S_2$ functions. C) Deformation field when displacement is expanded in terms of the neural network along with the corresponding similarity loss function. Red Dots indicate points with negative Jacobian. D) the same outputs when NODE is employed, showing the resulting transformation is physically meaningful ($J>0$ everywhere) (Color figure online)

It is worth mentioning that although there are popular and fast methods in the literature, the resulting transformation is not necessarily invertible (e.g., Balakrishnan et al. (2019)), which is in contradistinction with the admissible deformation field in classical continuum mechanics. Similar to the previous example, we use an analytical format of $S_1$ and $S_2$ (see Fig. 3-B). Fig. 3-C shows the resulting transformation for neural network and NODE architectures for the entire domain of integrals and not only $\mathscr {B}^T$. The corresponding loss functions during training for both regular neural networks and the NODE have also been reported in Fig. 3-D. In Fig. 3-C, we indicated points with negative Jacobian with red color. While the transformation map for the NODE structure generates positive Jacobian for the entire domain, there are points inside of the whole with negative Jacobian in the map attained from the regular neural network. This example confirms that in contrast to regular neural networks that are not necessarily invertible, the NODE is a good candidate for invertible transformations, although it is computationally more expensive in the back-propagation step as well as in the calculation of the deformation gradient. Similarly to the previous example, we computed the Dice score for the entire domain, which is *0.986* for the NODE method.

**Fig. 4:**
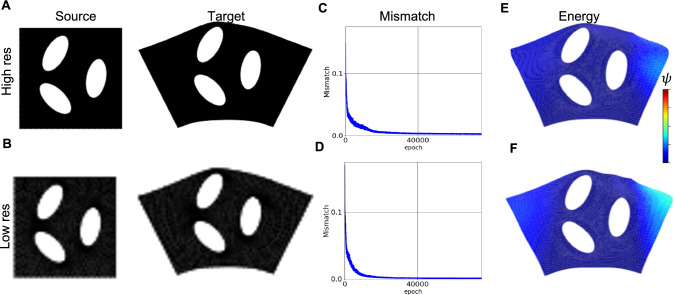
Resolution of image (discretization of source and target functions) impacts registration quality: A) source and target high-resolution images. B) source and target low-resolution images. C & D) mismatch energy loss for high- and low-resolutions, respectively. E & F) strain energy field corresponding to high- and low-resolutions

Using the next synthetic example, we intend to illustrate how the resolution of image data might affect the registration problem. In this regard, we analyzed the bending of the plate containing three ellipsoidal holes. To consider the effect of the resolution, we solved the problem for two sets of data: images with high resolution, which have been defined by using a fine mesh discretization, and low-resolution images that are $90 \times 90$ pixels. Neo-Hookean material has been selected for the constitutive equations. In this example, we performed the registration by selecting $\beta =1/6000$, with a total of 75000 epochs for the predictor. The resulting strain energy (i.e., $\psi$) for the predictor solutions has been shown in Figs. 4-E & 4-F, respectively, for high- and low-resolution data. From these figures, it is clear that the resolution may affect the final results. In particular, the computed Dice scores for the entire domain are *0.972* and *0.981* for the low and high resolutions, respectively. Additionally, the figure shows that a lower elastic energy level was achieved in the solution corresponding to the high-resolution data.

**Fig. 5:**
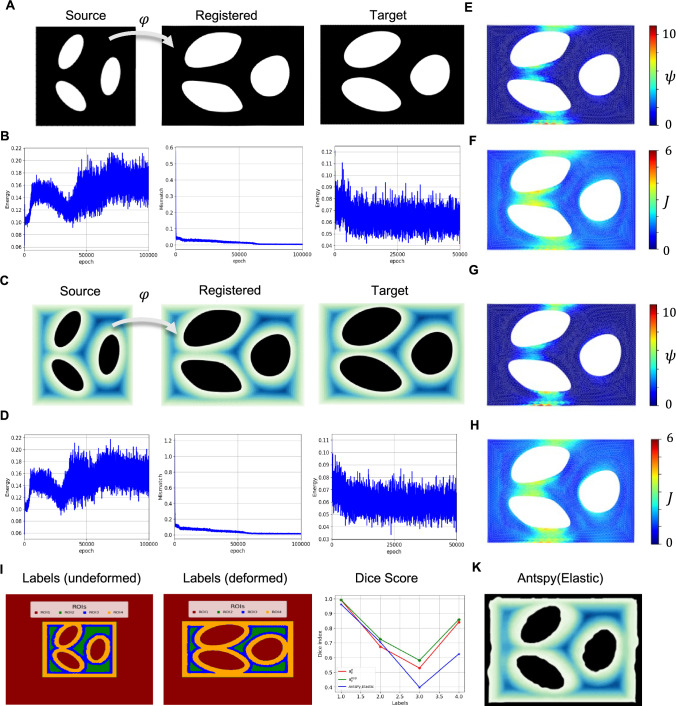
Gray scale or RGB information improve registration compared to binary approaches: A) Source, registered, and target images for the binary dataset. B) Mismatch energy, strain potential energy loss for predictor, and strain potential energy loss for corrector pertinent to binary datasets. C) Source, registered, and target images for the RGB dataset. D) Mismatch energy, strain potential energy loss for predictor, and strain potential energy loss for corrector pertinent to RGB datasets. E & F) Strain potential energy & Jacobian fields resulting from the binary dataset analysis. G & H) Strain potential energy & Jacobian fields resulting from the RGB dataset analysis. I) Initial and deformed configurations of a synthetic segmentation, along with the resulting Dice indices for both binary and RGB cases, and K) the registered configuration through AntsPy Elastic

The last synthetic example is employed to understand how more information in image data can change the results of a registration problem. In this regard, we solved the extension of a neo-Hookean plate with three ellipsoidal holes. We analyzed the problem using two image datasets, each with a size of $512 \times 512$ pixels: 1) Binary images containing information about the boundary points ( Fig. 5-A), and 2) RGB images having information about the interior points in addition to boundary points ( Fig. 5-C). To create the RGB images, we plotted $D(\mathbf{{X}})$. This format provides us with more information about the transformation of interior points. In addition, to measure the accuracy of the registration, we created undeformed and deformed versions of a synthetic labeled image as shown in Fig. 5-I. Furthermore, we compared the results of the registration in terms of the Dice index with AntsPy Elastic (xxx, yyy), which is a composition transformation of affine and deformable maps.

Fig. 5-A and 5-C illustrate the results for binary and RGB registrations, respectively. From these figures, one can notice that in terms of the outer boundary, both image data sets lead to almost the same accuracy, better than AntsPy Elastic (Fig. 5-K). Moreover, Figs. 5-B and 5-D indicate similar minimization paths for both datasets in both the predictor and corrector parts. However, as can be seen in Fig 5-I, the map resulting from the RGB data set has obtained better dice indices compared to those of binary and AntsPy Elastic. This result implies that when the image pair has more information, a better registration can be expected. Also, we have shown energy and Jacobian fields relevant to binary and RGB data in the corrector solutions in Figs 5-E to 5-H, which are almost the same for both data sets. Hence, in general, better geometrical registration can be achieved when more information is given in the dataset, as expected in medical image analysis.

### Biological Examples

In this part, we apply the new method to address practical questions. In particular, we utilized the method to quantify mechanical stresses during the regeneration of a zebrafish tail, brain shrinkage induced by cerebral atrophy associated with normal brain aging, and cortical expansion during fetal brain development during gestational weeks 21 through 36. However, before introducing the examples with growth, we start by showcasing a benchmark registration problem relevant for medical image analysis without growth biophysics.

#### Benchmarking Brain Registration

Prior to considering the coupling of growth and deformation with our method, we first show that the method is able to obtain high-quality registration for a complex benchmark such as brain registration, which had received widespread attention in the literature (Toga & Thompson, 2001; Hernandez et al., 2009; Lombaert et al., 2014). In this regard, we utilize NIREP data, a publically available data set, that has been skull-stripped and annotated to a total of 33 regions of interest (ROIs) by neuroradiologists (for more information, we refer the reader to Christensen et al. (2006)). The MRI data, along with the segmented nifty files, can be found in https://github.com/andreasmang/nirep. We intentionally showcase two brain scans that exhibit substantial differences with respect to intracranial volume and orientation of the brain to assess how effectively the new setup can register them. To that end, we selected images **na10.nii.gz** and **na02.nii.gz** as the initial and target images, respectively. we selected $\beta =0.0001$.

**Fig. 6:**
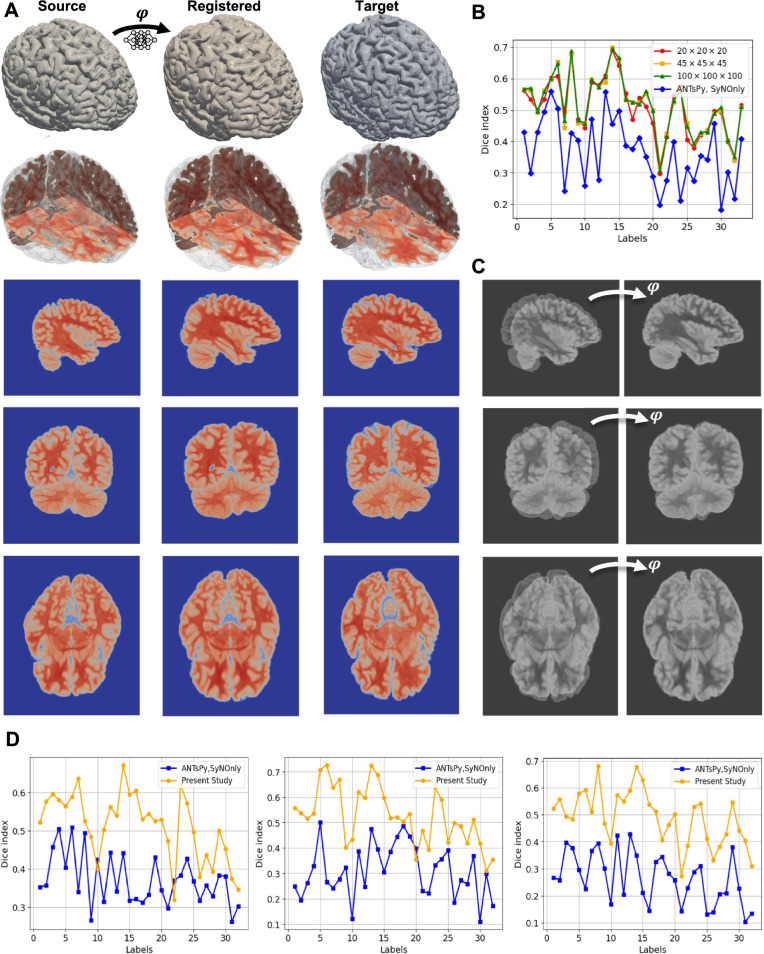
Benchmark problem for brain registration. A) Iso-surfaces along with sample sagittal, coronal, and axial cross sections corresponding to source, registered, and target of NIREP MRI datasets MRIs (na10.nii.gz to na02.nii.gz). B) Comparison of registration methods in terms of the Dice index between the current method across different discretization sizes and ANTsPy (SyNOnly) for all ROIs in the dataset. C) Overlay of initial and target intensities as well as the target and deformed geometry from the registration process, showing good alignment was achieved. D) Comparison of the result of registrations for three more pairs chosen from NIREP data sets(left: na16.nii.gz to na05.nii.gz, middle: na11.nii.gz to na03.nii.gz, and right: na12.nii.gz to na01.nii.gz)

The different views of the brain have been plotted to show the quality of the registration. In particular, Fig. 6-A summarizes the results of the problem by including a 3d view of the initial, registered, and target MRIs along with some random selection of the corresponding sagittal, coronal, and axial cross sections. For better comparison, we also included the merged version of cross sections in Fig. 6-C. The results show a good alignment between the warped-out initial and target images. We also quantitatively compared the result of our method with that of xxx (yyy), a Python library based on ANTs, one of the most commonly used registration algorithms for brain data (Klein et al., 2009; Ou et al., 2014). Both methods were compared based on the Dice similarity coefficient defined in (4.5). As previously stated, the NIREP data contains segmented data, a total of 33 labels. Thus, we evaluated (4.5) for each label and reported the results alongside those obtained using the method "SyNOnly" in Fig 6-B. This result suggests that our new method produces comparable registration results in comparison to one of the well-established algorithms widely used by the neuroscience community. Additionally, we analyzed the effect of discretization in the predictor calculation by considering grids of $20 \times 20 \times 20$, $45 \times 45 \times 45$, and $100 \times 100 \times 100$, which demonstrates the convergence of the calculation.To further ensure the independence of the comparison from the choice of samples, we repeated the same calculation for three pairs of datasets, which shows a similar trend in the quality of registration.

**Fig. 7:**
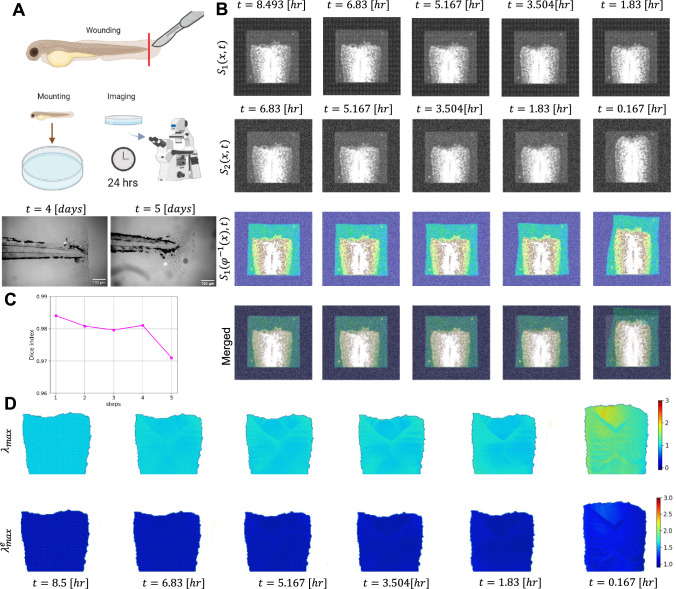
Zebrafish tail wound. A) A schematic view of the experimental steps carried out to obtain the dataset and focal images corresponding to four and five days after the amputation. B) Initial ($S_1(x,t)$), target ($S_2(x,t)$), and registration results ($S_1(\varphi ^{-1}(x),t)$) have been represented in the first, second, and third rows, respectively, while the merged of them have been demonstrated in the fourth one. C) Dice score for all steps of the registration. D) Comparison of the total and elastic deformation gradients in terms of maximum principal stretches after applying the corrector step with growth dissipation

#### Growth Examples

In the first example, we perform an analysis of the regrowth of the zebrafish tail. After amputation, the tail starts growing, and the exact signals that activate progenitor cells to cue regeneration are unknown. A hypothesis, in this regard, is that the mechanical forces within the tissue cue regeneration. To explore this assumption, it is required to approximate the spatial distribution of deformation over the tissue given a series of images of the regenerating tail. Physics-based registration can thus provide a better insight into this process. Experimentally, we cut the tail using a scalpel and imaged the fish for 24 hours using a cell cycle indicator (Sugiyama et al., 2009) to see where replication is initiated during the process of regrowing lost tissue (see Fig 7-A).

For registration analysis, we followed the sequential approach mentioned above. In this regard, we considered a total of six frames from the image data (i.e., frames at 10 min, 1hr 50 min, 3hr 30 min, 5hr 10min, 6hr 50 min, and 8hrs 30 min) in such a way that the selected images capture the main deformation over 24 hrs. We performed the analysis in a time-reversed manner for simplicity. Specifically, in our sequential approach, we started from frame 6 and sequentially returned to frame 1. Our reasoning for doing so is to counteract the limitations of the experimental dataset, in which the majority of the initial deformation happens within the first fifteen minutes. Because the tail fin relaxes gradually from the peak of that deformation over the course of many hours, correctly mapping the movement of the tissue from the relaxed to the contracted state by starting with a later shape and working backward through a slower shape change was more intuitive than attempting to derive the entire tissue movement from a very fast initial contraction. In the predictor part, we trained for 50000 epochs overall, with $\beta =0.1$. For the corrector part, we trained the first three steps for 50000 epochs each and the last two steps for 100000 epochs each. We also set initial growth parameters as follows: $\kappa =-17,-17,-17,-11,-5$ for the first to fifth steps, respectively. Fig 7-B shows the result of the registration. The first two rows in Fig 7-B shows directly the image data obtained from experiments, while the third row is the result of the registration, i.e., the deformation of the initial frame into the shapes of the subsequent frames. To facilitate clearer observation, we merged the second and third rows and reported in the fourth one. As can be seen a good registration obtained in all steps. To show this fact, the dice score across the entire domain for each step of registration has been plotted in Fig. 7-C.

The key outcome from the registration is, however, not the alignment between data and deformed images but the implications for tissue growth. The first row of Fig 7-C shows the contours of the maximum principal stretch after running the corrector to satisfy mechanical equilibrium. Initially, the deformation is minimal, which is intuitive since the images do not seem to indicate large shape changes over the last 6 hrs. However, in the last two time frames, between the first two hours, from 10 minutes to 1 hour 50 minutes, there is significant deformation, with larger stretches toward the tip of the regenerating tail. Also depicted in Fig 7-C are the elastic contributions to the deformation. Recall that the corrector also allows for tissue growth to dissipate mechanical energy. If the deformation were fully plastic, that is, if growth were able to completely absorb the shape changes, then the resulting elastic stretch contour would be 1. In reality, Fig 7-C shows that while growth allows for most of the energy to be dissipated, the resulting elastic deformation is not exactly 1 but rather varies slightly with respect to 1. In the last frame of Fig 7-C, it can be seen that the largest elastic deformation is expected at distal end of the regenerating tail. We anticipate that this new information will shed light on the biological mechanisms regulating zebrafish tail wound healing.

**Fig. 8:**
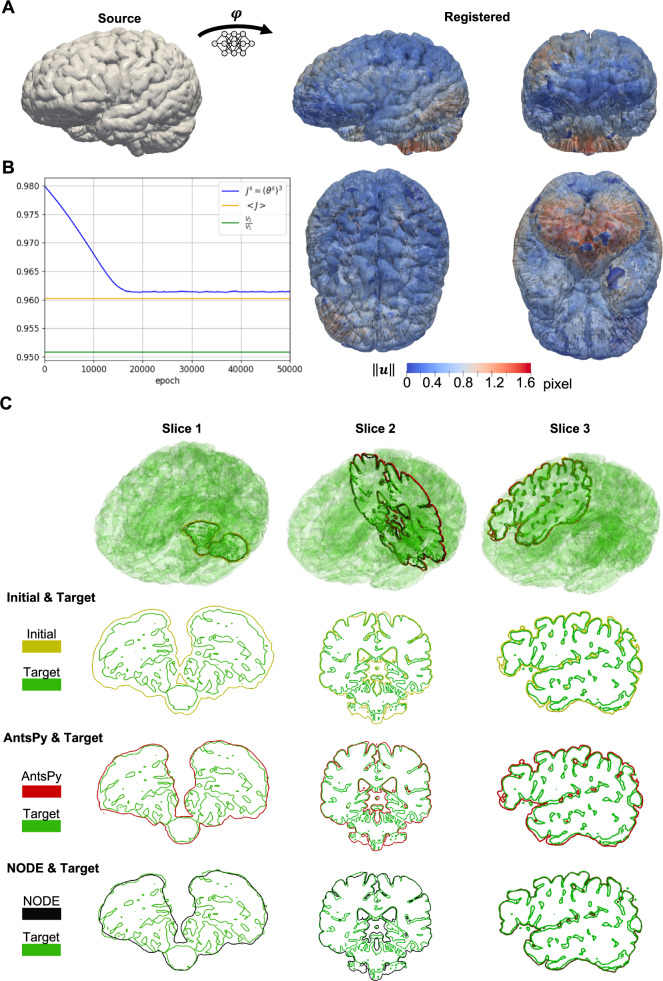
Brain atrophy example. A) Iso-surface of the baseline and the follow-up obtained from registration along with quiver plots of displacement fields on selected points. The direction of the arrows confirms the contraction of the brain. B) The convergence of the new solution procedure in the corrector part. The major part of contraction is due to permanent shrinkage, $J^s=(\theta ^s)^3=0.961431$, denoting a reduction of 5%. C) Comparison of the registration between the present study and ANTsPy (SyNOnly) in terms of the iso-surface of the brain with zoomed-in slices

In the next biological case, we quantify brain volume loss in a cognitively subject between the ages of 71 and 75 whose scans were obtained from the Alzheimer’s Disease Neuroimaging Initiative (Jack et al., 2008; Petersen et al., 2010). In this example, we are primarily interested in brain deformation, and not just registration between images alone. To that end, we first used ANTsPy to rigidly align the source and target image. Then, we used our method while prescribing homogeneous and isotropic shrinkage, i.e., negative growth, as detailed in Section 3.3. In our analysis, we optimized for the (negative) growth rate $\kappa$ for the given MRI data while maintaining equilibrium. Growth is optimized such that elastic energy is dissipated. Because this deformation is shrinking or negative growth rather than positive growth or addition of mass, we switch the notation from $\theta ^g$ to $\theta ^s$, but this is just a slight change in notation, the split of the total Jacobian is the same as Eq. (3.8), $J=J^eJ^s, J^s = (\theta ^s)^3$. Fig. 8-A shows multiple views of the brain’s displacement magnitude field as well as a limited number of nodal displacement vectors that illustrate how the brain deforms. We clearly observe a uniform contraction of the cortical surface representative of brain shrinkage. Our method allows us to quantify the degree of volume loss, as shown in Fig. 8-B. As can be seen, there is a five percent permanent reduction in volume between the baseline and follow-up scan $\kappa =0.013111, J^s=(\theta ^s)^3=0.961431$, which accords with the average value of the total Jacobian, i.e., $<J>\approx 0.96$. Also, it is observable that a good convergence of the shrinkage parameter was achieved, implying that the major part of the deformation is due to permanent brain shrinkage, with only a small amount of residual elastic deformation left. As shown in Fig. 8-B, the major deformation occurs in cerebellum. Therefore, to assess the quality of geometric registration, we intentionally selected a slice of the brain in this region and compared the results with ANTsPy, as illustrated in Fig. 8-C. We present results for three zoomed-in slices: one from the cerebellum and two randomly selected. For each slice, we provide the corresponding target image and the results obtained using ANTsPy (SyNOnly). To facilitate comparison, we separately compare each slice by presenting the initial image, the results from ANTsPy, and the results from the present method against the target image. As observed, the geometric registration obtained using the NODE method is comparable to the results achieved with ANTsPy. Additionally, the resulting Dice score for the entire geometry is *0.965*, indicating a good registration.

**Table 1:** Central parameters for ablation study

**Steps**	**Architecture**	**Time Step**	**Regularizing Term**	**Strain Potential**
Predictor	[2,40,40,40,2]	${\nicefrac {1}{15}}$	$R = {\left\| {\textbf{F}} \right\| ^2} + {\left( {\log J} \right) ^2}$	N/A
Corrector	[2,40,40,40,2]	${\nicefrac {1}{15}}$	N/A	(4.3)

**Fig. 9:**
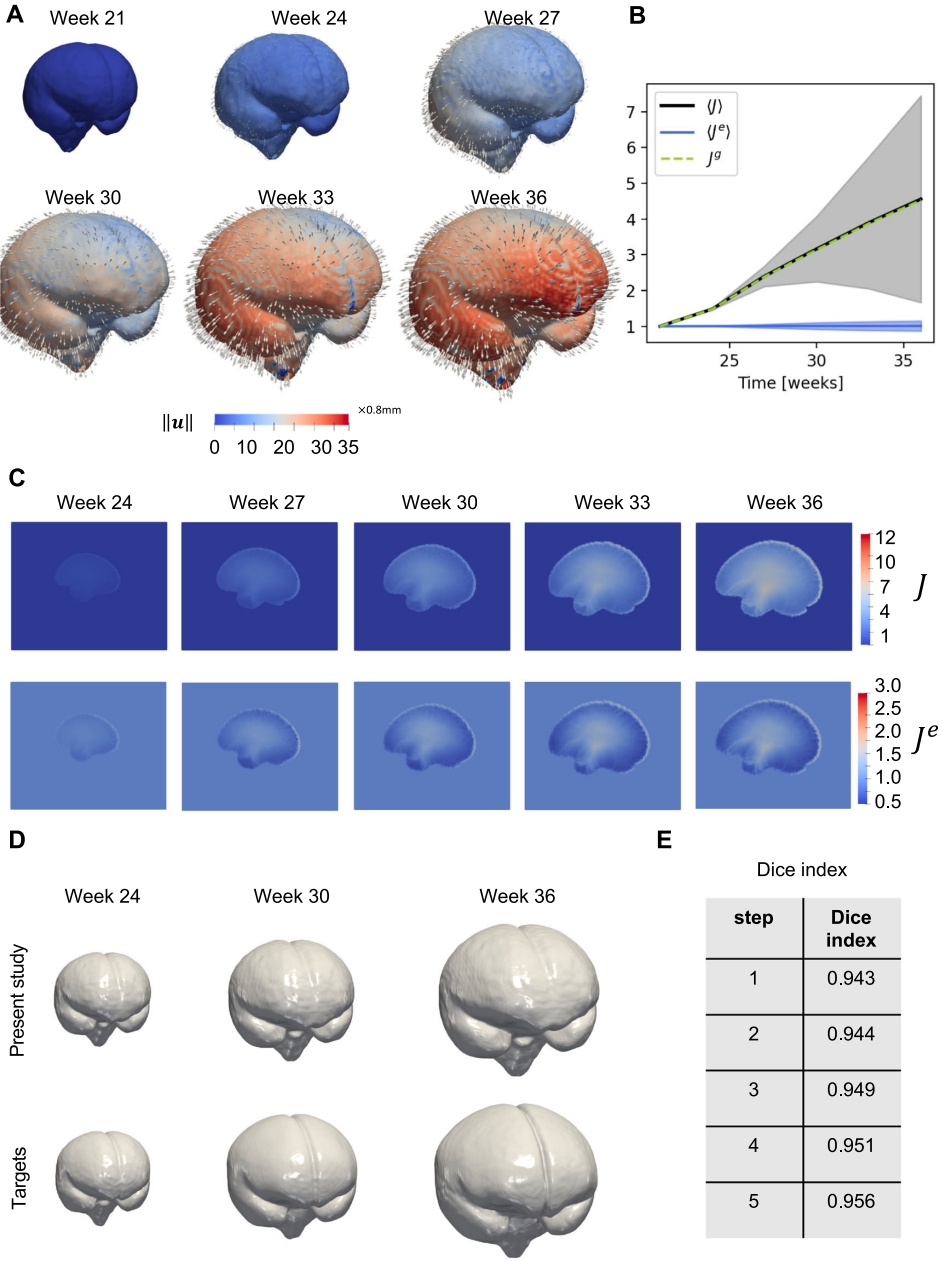
The extensive cortical expansion associated with early fetal brain development. A) Brain iso-surfaces for all six steps together with quiver displacement fields. B) statistical representation of the Jacobian field for total, elastic, and growth parts. The average growth Jacobian is closely equal to the total Jacobian for all steps, while the elastic Jacobian remained close to unity. C) Comparison of the total and elastic Jacobians for a given sagittal cross section for all steps, confirming the deformation is mainly non-elastic, and the growth deformation is able to dissipate most elastic energy and residual stresses. The results suggest that growth rather than elastic deformation is likely the cause of the large shape and volume changes during embryonic development. D) Comparison of three steps of the registration between the present method and the AtnsPy(SyNOnly). E) The dice score indices corresponding to registration of the whole geometry of the brain

For the last biological example, we chose to study the extensive cortical expansion associated with early fetal brain development. To that end, we analyzed the unbiased, deformable, spatiotemporal atlas of the fetal brain provided by The Computational Radiology Laboratory at Harvard University (Gholipour et al., 2017). The atlas consists of weekly images between gestational weeks 21 and 37. To demonstrate the capabilities of the algorithm, we selected the images three weeks apart, i.e., weeks 21, 24, 27, 30, 33, and 36. Analogous to the zebrafish example, we implemented the sequential version of the method. We used $\beta =0.1$ and 50000 as the epoch number in the predictor part. In the corrector part, to obtain a good convergence for the growth parameter, we considered 100000 epochs in the first step and 50000 for others. Fig. 9-A shows the displacement magnitude fields and displacement vector field. We generally observe a relatively uniform expansion of the cortical surface. Additionally, early weeks are characterized by very similar displacement magnitudes across the whole surface, while later weeks exhibit increasingly heterogeneous magnitudes. Specifically, we see a localization of maximum displacement magnitudes in the frontal and temporal lobes. In Fig. 9-B, we statistically summarized the distribution of elastic, total, and growth Jacobian in the whole volume. As can be seen, the average Jacobian due to growth is the same as the average of the one relevant to total deformation during all weeks, while the average elastic Jacobian increases slightly more than unity as a function of time. Strikingly, the brain’s volume increases nearly 5-fold during the mid-to-late gestational stages. The total Jacobian (top row) and elastic Jacobian (bottom row) shown in Fig. 9-C indicate regions of highest volume growth. It is evident from the coronal sections that growth predominantly occurs in deep tissue structures as well as the outer cortical layer. This confirms that deformations during these gestational weeks can be explained by tissue growth, which can minimize the total elastic energy without inducing large residual stresses. Analogous to previous examples, we compared the registered images with target ones and computed the Dice score for all steps of registration, as shown in Fig. 9-D and Fig 9-E, respectively.

## Ablation Study

In this part, we present a comprehensive ablation and hyperparameter study of the proposed algorithm for the shear-contraction problem defined in Fig. 3. This problem is one that proves difficult for conventional deep neural networks and required the NODE architecture to guarantee invertivibility. Considering that the method has two major parts, i.e., the predictor and corrector, in which we approximate the deformation field with a flow algorithm (i.e., NODEs as the deformation approximator), we analyze the following: 1) for the predictor part, we consider the effect of different regularization terms, different values of hyperparameter $\beta$, the number of neurons of each layer for the core neural network, the number of its layers, and pseudo time steps $\Delta \tau$; 2) in the corrector part, we test different choices of regularizing terms and various $\beta$ assumed in the predictor, the number of neurons in each layer of the core neural network, the number of its layers, and the number of pseudo time step $\Delta t$. For varying the parameters we consider those in Table 1. Then, we vary the target parameter while keeping all other parameters fixed at their baseline values.

**Fig. 10:**
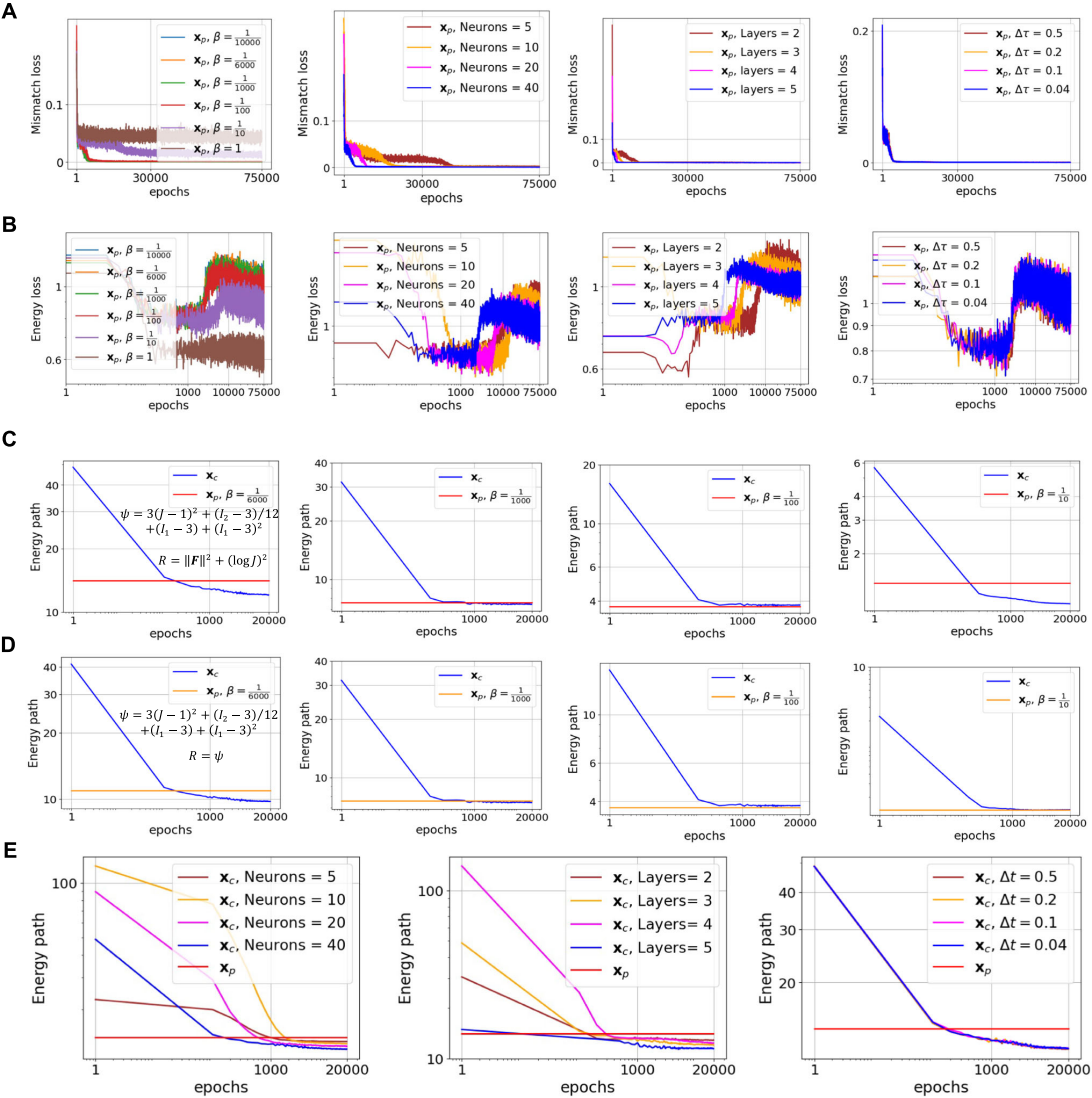
Ablation study for shear-constraction problem defined in Fig. 3: A) Mismatch loss versus epoch (log-log scale) in the predictor part for different values of $\beta$, the number of neurons, the number of layers, and pseudo time steps. B) Energy loss versus epoch (log-log scale) in the predictor part for different values of $\beta$, the number of neurons, the number of layers, and pseudo time steps. C) Minimization of energy paths in the corrector part for different values of $\beta$ when the regularizer is selected to be different from the strain potential energy. D) Minimization of energy paths in the corrector part for different values of $\beta$ when the regularizer is selected to be the same as the strain potential energy. E) Minimization of energy paths in the corrector part for different numbers of neurons, layers, and pseudo time steps with the reguralizer $R ={\left( {\text {log}}J\right) }^2+{\Vert \mathbf{{F}}\Vert }^2$

Figs. 10-A & -B show the mismatch loss and the elastic energy loss associated with the ablation study of the predictor part by varying $\beta$, the number of neurons, and the number of pseudo time steps. As mentioned in Pawar et al. (2022), the mismatch loss and the regularizing energy term are two competing factors balanced by $\beta$. Larger values of $\beta$ lead to a smooth mapping that partially minimizes the mismatch loss, while lower values result in effective registration but potentially loss of regularity. Hence, the value of $\beta$ should be small enough to achieve good registration while still resulting in a smooth transformation. The trade-off of the multi-objective optimization as a function of the hyper-parameter $\beta$ is also illustrated in the Appendix.

Furthermore, similar behavior is observed when changing the number of neurons and layers: the higher values for number of neurons and layers lead to faster and better convergence. However, this choice is accompanied by more computational costs during learning and back propagation. Hence, in this setting, it is required to strike a balance between the accuracy and computational costs. Changing the $\Delta \tau$ has less effect on the path of the minimization. However, as shown in the shear contraction example, the definition of the flow algorithm is crucial since it guarantees the invertibility of the resulting transformation.

**Fig. 11:**
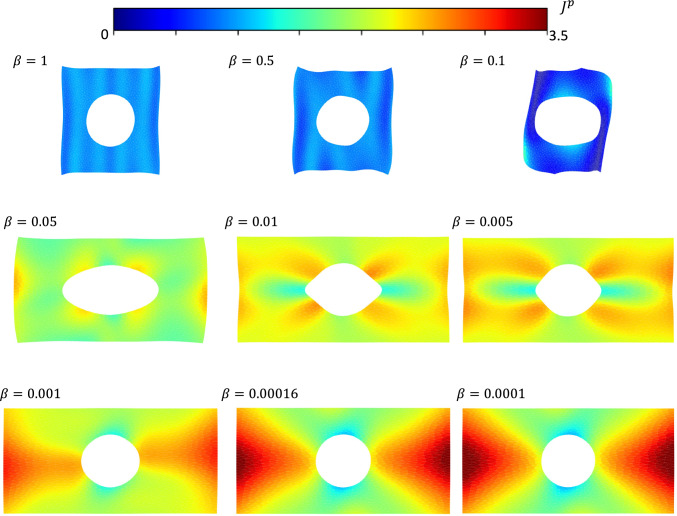
Effect of the hyperparameter $\beta$ on registration quality in the extension of the plate with a hole considered in Fig 2. As can be seen, this parameter should be selected small enough to achieve proper registration

**Fig. 12:**
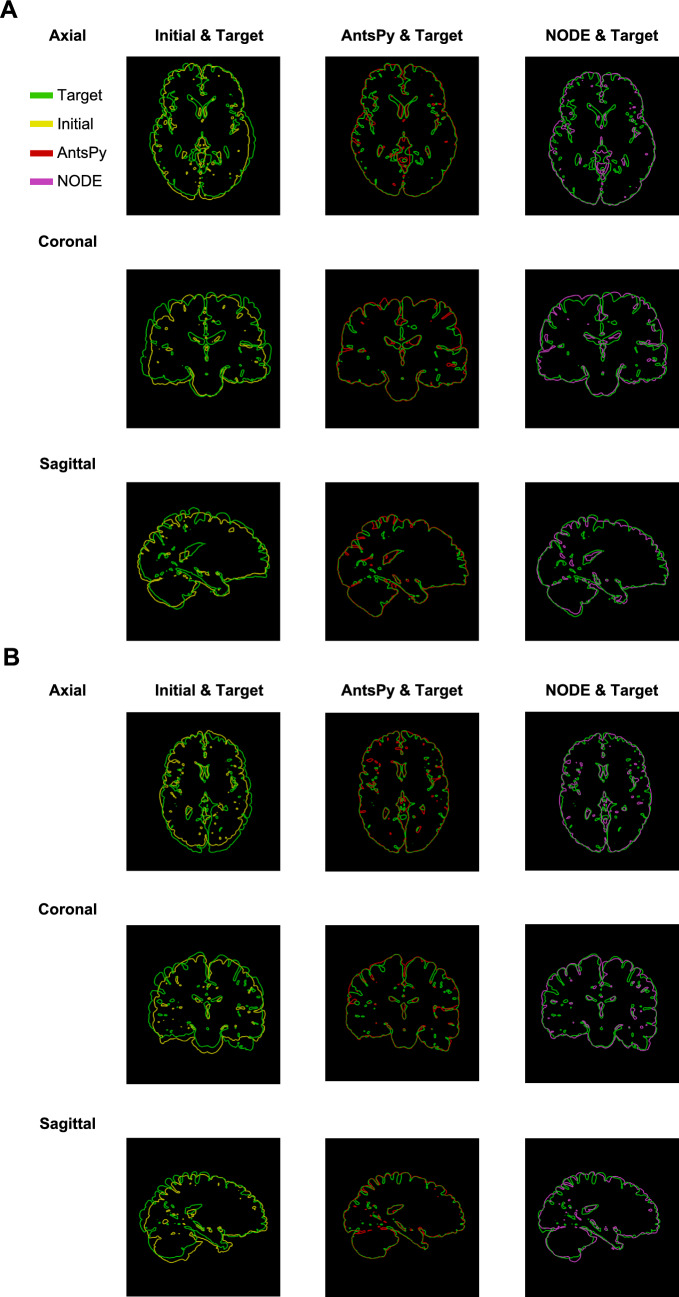

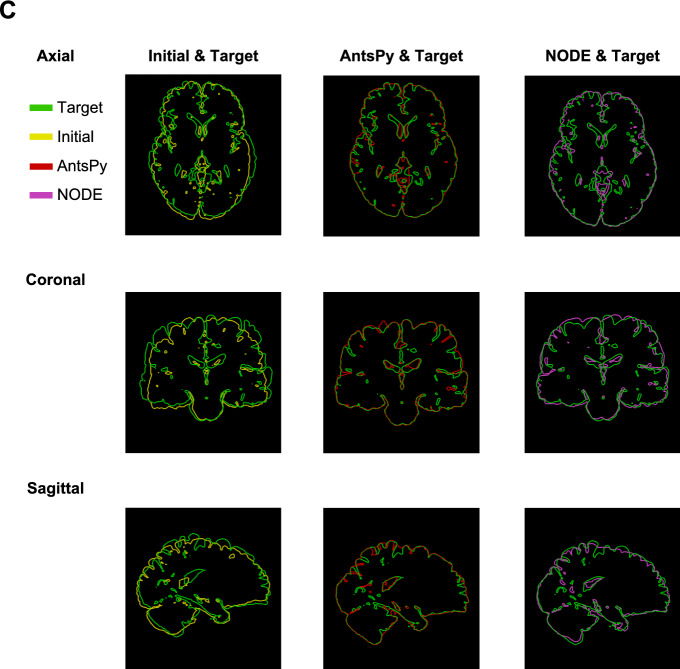
Comparison of registration obtained from the present method with those of ANTsPy for NIREP data: A) na16.nii.gz to na05.nii.gz, B) na11.nii.gz to na03.nii.gz, and C) na12.nii.gz to na01.nii.gz.

In the corrector part, depending on the choice of the regularizing term in the predictor, one expects different minimization paths in the corrector. This fact has been illustrated in Figs. 10 -C & -D. Fig 10-C shows the strain energy path minimization when the regularizing term is $R = {\left\| {\textbf{F}} \right\| ^2} + {\left( {\log J} \right) ^2}$, while Fig. 10-D indicates the minimization of the elastic energy by the corrector when the regularizing term is selected to be the same as the strain energy potential. As can be seen, the corrector part reached to a stationary point of total potential energy in all cases, but with different initial conditions given by the predictor. Also, Fig 10-D shows that when $\beta =\frac{1}{6000}$, the predictor path leads to a strain energy that is then further minimized by the corrector. In contrast, for $\beta =\frac{1}{1000}$, the corrector solution is close to the predictor result, implying the existence of some specific values of $\beta$ where the predictor solution may be close to satisfaction of the equilibrium equations. Nevertheless, the choice of $\beta$ such that it provides good registration and good minimization of the elastic energy is difficult to obtain and might not exist. In contrast, our predictor-corrector split provides robust registratoin as well as minimization of the strain energy (indicative of equilibrium), as shown in Fig. 10-D.

Fig. 10-E shows the results pertinent to sensitivity analysis of corrector solution with respect to the number of neurons, the number of layers, and $\Delta t$ have similar effect in the path of minimization of the corrector as in the predictor part. In this analysis, we selected the results corresponding to $\beta =\frac{1}{100}$ and $R = {\left\| {\textbf{F}} \right\| ^2} + {\left( {\log J} \right) ^2}$.

## Discussion and Conclusion

In this article, we presented a new algorithm for registering 2d and 3d images while concomitantly solving physics-based boundary value problems. The approach is composed of two parts: 1) predictor and 2) corrector. In the predictor, the algorithm minimizes the mismatch loss, while a regularizer is defined to preserve the smoothness of the resulting transformation. In the corrector, a Dirichlet boundary value problem is then defined and solved in a weak sense, similar to the finite element method. The boundary condition in the corrector is determined by the map obtained from the predictor transformation, which is preserved via distance functions. This corrector part results in finding a stationary (minimizer) point of the total potential energy, leading to a solution that fulfills the equilibrium equation of continuum mechanics in a weak sense. The predictor-corrector split crucially allows not just for a better result than multi-objective optimization registration methods (Pawar et al., 2022) but enables complex multi-physics coupling such as growth and remodeling (Ambrosi et al., 2011).

To show the plausibility of the method, we considered several synthetic and real biological examples. Specifically, by using synthetic examples, we showed that the integral format of a PINN (direct potential energy minimization) where the prescribed displacement boundary condition is satisfied exactly, obtains a solution close to the solution of the FEM. This fact is because the method follows the same paradigm as FEM, which is the minimization of the potential energy. In contrast, strong form or variational PINNs commonly impose boundary conditions via a penalty and result in multi-objective optimization, and it may result in a solution that only partially satisfies equilibrium and boundary conditions (Sukumar & Srivastava, 2022). The registration method, in particular the corrector part, satisfies a Dirichlet problem, i.e., it solves a problem with exact boundary conditions, where these boundary conditions are the result of the predictor. The boundary conditions are imposed through distance functions, a strategy also used in the broader context of PINNs to impose boundary conditions (Sukumar & Srivastava, 2022). In contrast to the usual PINN problems, the registration problem is inherently ill-posed as there are infinitely many predictor solutions that vanish the mismatch loss but define a different Dirichlet problem for the corrector. Nevertheless, this characteristic is not considered an anomaly but rather lies in the nature of registration problems.

Among existing deep learning architectures proposed for image registration (see, for example, Balakrishnan et al. (2019)), we showed that the NODE is a good candidate to produce invertible transformations while having strong expressibility. It is worth mentioning that other invertible architectures, such as NICE (Dinh et al., 2014) and Real NVP (Dinh et al., 2016), were tested. Still, obtaining a good deformation field was challenging, which can be attributed to the predesigned invertible architectures of the main building block of such methods. We further observed that the resolution of the image may result in different solutions. Hence, input data with high resolution leads to better registration. By performing registration on binary and RGB images, we also noticed that better registration may be obtained when the image has more information in terms of intensity.

To benchmark the method against relevant applications, we applied it to perform the registration of brain MR images. In this regard, we selected two MRI data with noticeable differences from NIREP data (Christensen et al., 2006), conducted the registration (only predictor part), and compared the results with ANTsPy (Avants et al., 2009a, b). The results indicate that the NODE structure is comparable and can even outperform state-of-the-art.

The main motivation of the proposed method is to go beyond registration and demonstrate the ability to solve biophysics problems within the same framework, which typically involve complex mechanics and multi-physics coupling and additional external software (López et al., 2023; Min et al., 2023; Wang et al., 2020).

In particular, in this paper, we contribute to the field by introducing the predictor-corrector split, which gives us much more flexibility for the types of multi-physics problems we can tackle while retaining the same deep learning framework. Three biological examples with tissue growth were considered to underscore the unique abilities of the proposed approach. Firstly, through the sequential format of the new technique, we managed to map total, elastic, and growth deformations across zebrafish wound healing. Additionally, we modeled brain atrophy. The problem of fetal brain development was also studied. The algorithm was able to find a continuous deformation map from the geometry at week 21 up to week 36 during pregnancy. The volume changes observed in this time frame are substantial, with total Jacobians averaging above 4-fold volume changes. Thus, the method can handle extreme deformation. Growth biophysics explains that most of the deformation can be attributed to growth, leading to the dissipation of mechanical energy.

The biological examples contain several assumptions regarding the material behavior, growth ODE form, growth tensor form, and assumption of homogeneous material. Each of these problems can be further refined in future work to account for the complex biological mechanisms behind zebrafish wound healing (De Vos et al., 2017), brain atrophy in neurological disease (Blinkouskaya et al., 2021; Blinkouskaya & Weickenmeier, 2021), and fetal brain development (Wang et al., 2020). Furthermore, in this work, we enforced the physics with a method similar to variational PINNs, while the method of the neural operator networks can be adapted, which generalizes the solution to other PDEs (He et al., 2024, 2024). This is an interesting avenue of future research when the PDE is unknown or to accelerate the solution in the case that similar geometries are processed, e.g., in the case of brain atrophy (Visser et al., 2023). Extension to thin membranes is also a problem of interest for medical image analysis we intend to tackle (Pouch et al., 2014, 2020). Moreover, in this study, we demonstrated the application of the new method by testing it on a few brain examples. However, to establish statistically significant knowledge regarding brain biophysics, the new method should be applied to larger datasets (Marcus et al., 2007; Ou et al., 2014).

Furthermore, the ablation study provided deeper insights into the parameters of the new algorithm. The numerical results indicated that altering the number of neurons and layers in the neural network has a similar effect on the accuracy of the method in both the predictor and corrector, suggesting an optimal architecture to balance computational cost and accuracy. Additionally, the ablation study further confirmed that, unlike other methods, our proposed approach can obtain the stationary point of the total potential energy similar to the finite element method in the corrector step, regardless of the chosen regularizing term. Hence, as a future work, it is desirable to develop a hybrid method where the predictor part is done with current registration methods available in literature while the corrector part is carried out by the present algorithm. The key requirement for this approach is ensuring the invertibility of the predictor.

We investigated the computational cost of the method with two predictor cases, both with epoch=50000. For a 2d analysis, which we performed on the plate extension with a hole, the computational cost was about an hour with Nvidia A100 Tensor Core GPU (80 GB). For a 3d problem, which we performed for the predictor of shrinkage problem, we observed a computational cost of 3 hrs with Nvidia A10 Tensor Core GPU (24 GB). The major computational cost can be attributed to the NODE’s training. However, as illustrated in Fig 3, obtaining a general function based on an evolutionary first-order dynamical system is a rich structure, although it might be computationally demanding. A strategy that can reduce the computational cost of the method, which we plan to implement in future work, is to adopt an adaptive scheme by applying an early stop algorithm. In particular, one can provide a criterion that prevents the algorithm from further training when the desired convergence is achieved. Another strategy, in this regard, is by employing refinement strategies to sample in regions with higher error, similar adaptive re-meshing in finite element or spline-based registration (Pawar et al., 2016). Finally, since the displacement field is constructed from the (pseudo) velocity field, the order of the problem can be reduced by leveraging reduced-order methods, such as principal component analysis, which allows it to be solved with fewer degrees of freedom.

As the theory and numerical results suggest, the new method is robust. In particular, the geometric registration in the predictor part is comparable with existing methods. In addition, the new solution procedure attaches physics to the underlying transformation by finding the minimizer of the total potential energy for an equivalent Dirichlet problem in the corrector part. The predictor-corrector split enables rich biophysics modeling in the corrector portion, for example, modeling tissue growth, a hallmark phenomenon of living matter. We thus anticipate that this method can be widely used in various applications, especially medical image analysis.

## Data Availability

The code to replicate this manuscript’s example is available at https://github.com/AHA-H1987/NODE_Registration.

## Appendix

Since the calculation of the deformation gradient is required in the new method, we present an approach for computing it in this section. The approach is the consecutive application of the chain rule that can be implemented concurrently with the NODE calculation. This method can be particularly effective when a small (pseudo) time step is necessary to produce an invertible transformation, as it circumvents the one-time calculation of automatic differentiation for the entire forward Euler method employed in NODE calculation.

For the predictor, one can compute the deformation gradient alongside of (3.3) as follows:

A.1 $$\begin{aligned} \begin{aligned}&{\textbf{F}}_p^{(i + 1)} = \left( {{\textbf{I}} + \Delta \tau \,{\nabla _{{\textbf{x}}_p^{(i)}}}{\textbf{NN}}({\textbf{x}}_p^{(i)};{{\textbf{w}}_p},{{\textbf{b}}_p})} \right) {\textbf{F}}_p^{(i)},\,\,\,\, \\&\quad i = 0,...,{n_p},\,\, \Delta \tau = \frac{1}{{{n_p}}},\\&\quad {\textbf{F}}_p^{(0)} = {\textbf{I}}, \end{aligned} \end{aligned}$$

where ${{\nabla _{{\textbf{x}}_p^{(i)}}}}$ denotes the gradient of the neural network with respect to ${{\textbf{x}}_p^{(i)}}$, which can be carried out with automatic differentiation. Apparently, the calculation of (A.1) can be carried out concurrently with (3.3).

In the corrector part, a similar recursive equation can be obtained in parallel with (3.6):

A.2 $$\begin{aligned} \begin{aligned} {\textbf{F}}_c^{(i + 1)}&= {\tilde{\textbf{F}}}_c^{(i)}{\textbf{F}}_c^{(i)},\,\,\,i = 0,...,{n_c},\,\,\,\,\Delta t = \frac{1}{{{n_c}}},\\ {\tilde{\textbf{F}}}_c^{(i)}&= {\textbf{I}} + \Delta \tau \,{\nabla _{{\textbf{x}}_c^{(i)}}}{D_p}({\textbf{x}}_c^{(i)};{\textbf{x}}_{{\mathscr {B}^I}}^{p(n)})\\&\quad \otimes {\textbf{NN}}({\textbf{x}}_p^{(i)};{{\textbf{w}}_p},{{\textbf{b}}_p}) + {D_p}({\textbf{x}}_c^{(i)};{\textbf{x}}_{{\mathscr {B}^I}}^{p(n)})\\&\qquad {\nabla _{{\textbf{x}}_c^{(i)}}}{\textbf{NN}}({\textbf{x}}_c^{(i)};{{\textbf{w}}_c},{{\textbf{b}}_c}),\\ {\textbf{F}}_c^{(0)}&= {\textbf{F}}_p^{({n_p})},\,\,{\tilde{\textbf{F}}}_c^{(0)} = {\textbf{I}}, \end{aligned} \end{aligned}$$

where $\otimes$ stands for the dyadic product.

## Appendix B

In this part, the tuning process of the hyperparameter $\beta$ for the extension of the plate with a hole (see Fig. 2) is shown. To ensure the convergence of the mismatch loss for all values of $\beta$, the number of epochs was set to 100,000 for all cases. As can be seen in Fig 11, the large values of $\beta$ lead to almost zero deformation while the quality of the registration improves with the reduction of $\beta$, where good registration is obtained by setting $\beta =\frac{1}{6000}$.

## Appendix C

In this section, we additionally present axial, coronal, and sagittal views of the registration results for the three image pairs used to generate the results in Fig. 6-D. As observed, the results of the proposed method are comparable to those of ANTsPy.
